# Outbreak of invasive meningococcal disease caused by a meningococcus serogroup B in a nursery school, Wallonia, Belgium, 2018

**DOI:** 10.2807/1560-7917.ES.2022.27.9.2100224

**Published:** 2022-03-03

**Authors:** Stéphanie Jacquinet, Wesley Mattheus, Sophie Quoilin, Chloé Wyndham-Thomas, Charlotte Martin, Dimitri Van der Linden, André Mulder, Julie Frère, Carole Schirvel

**Affiliations:** 1 Epidemiology of infectious diseases, Department of Epidemiology and Public Health, Sciensano, Brussels, Belgium; 2 National Reference Centre for *Neisseria meningitidis*, Sciensano, Brussels, Belgium; 3 Infectious Diseases Department, CHU Saint-Pierre, Université Libre de Bruxelles (ULB), Brussels, Belgium; 4 Pediatric Infectious Diseases, General Pediatrics, Pediatric Department Cliniques universitaires Saint-Luc, Brussels, Belgium; 5 Institut de Recherche Expérimentale et Clinique (IREC), UCLouvain, Brussels, Belgium; 6 Pediatric Intensive Care Unit, Centre Hospitalier Chrétien (CHC) Liège, Belgium; 7 University Department of Paediatrics, Centre Hospitalier Universitaire (CHU) Liège, Belgium; 8 Agence pour une vie de qualité, infection prevention and control, Wallonia, Belgium

**Keywords:** Meningococcus B, outbreak management, prolonged outbreak

## Abstract

Although most invasive meningococcal disease (IMD) cases are sporadic without identified transmission links, outbreaks can occur. We report three cases caused by meningococcus B (MenB) at a Belgian nursery school over 9 months. The first two cases of IMD occurred in spring and summer 2018 in healthy children (aged 3–5 years) attending the same classroom. Chemoprophylaxis was given to close contacts of both cases following regional guidelines. The third case, a healthy child of similar age in the same class as a sibling of one case, developed disease in late 2018. Microbiological analyses revealed MenB with identical finetype clonal complex 269 for Case 1 and 3 (unavailable for Case 2). Antimicrobial susceptibility testing revealed no antibiotic resistance. Following Case 3, after multidisciplinary discussion, chemoprophylaxis and 4CMenB (Bexsero) vaccination were offered to close contacts. In the 12-month follow-up of Case 3, no additional cases were reported by the school. IMD outbreaks are difficult to manage and generate public anxiety, particularly in the case of an ongoing cluster, despite contact tracing and management. This outbreak resulted in the addition of MenB vaccination to close contacts in Wallonian regional guidelines, highlighting the potential need and added value of vaccination in outbreak management.

## Background

Invasive meningococcal disease (IMD) is a serious life-threatening illness and, in 2017, had a case fatality rate of 9.7% in the European Union/European Economic Area (EU/EEA) countries. Data from several high-income countries has shown a severe long-term burden of disease with major sequelae in 10–20% of survivors [1-4]. IMD is caused by *Neisseria meningitidis*, a Gram-negative bacterium present in the nasopharynx of healthy carriers, which is transmitted through droplets of respiratory or throat secretions. The disease mainly affects infants (< 1 year) and young children (1–4 years). Although IMD remains relatively rare in the EU/EEA, with notification rates of 0.6 cases per 100,000 population in 2017 [1], its severity and outbreak potential make it a major public health problem. Of the 12 serogroups, A, B, C, W, Y and X cause most IMD cases worldwide [5]. Despite decreasing trends, serogroup B remains the predominant serogroup in the EU/EEA, accounting for over 50% of cases overall in 2017 and for 70% of cases in children under 5 years of age [1].

In Belgium, IMD cases are under mandatory reporting to regional health authorities and the epidemiology in the country is in line with the EU/EEA trends. Since 2010, the estimated annual incidence has been 0.96 cases/100,000 population with a highest incidence rate in children under 5 years of age (5.4/100,000), and a predominance of serogroup B (ca 50% of cases) [6].

Although the vast majority of IMD cases are sporadic without identified transmission links, outbreaks, i.e. multiple cases of the same serogroup arising in a defined population over a short time period, occur in ca 3.1% of cases [7]. In IMD clusters, the time interval between an index case and subsequent cases is generally less than 3 weeks, with a large majority seen within the first 7 days [8,9].

Since 2013, two protein-based vaccines have been licensed in Europe for the prevention of IMD by group B meningococci (MenB), namely the multicomponent meningococcal serogroup B vaccine 4CMenB (Bexsero, GSK Vaccines S.r.l., Siena, Italy) and rLP2086 (Trumenba, Pfizer, Brussels, Belgium). Only 4CMenB is licensed for use in children under 10 years of age. MenB vaccination, unlike meningococcal C (MenC) vaccination, is not included in Belgium’s free-of-charge child vaccination programs, but can be considered individually with a general practitioner or paediatrician.

### Outbreak detection

Here we describe a cluster of three IMD cases caused by a MenB strain in children aged 3–5 at a nursery school, with the cases spread over a 9-month period in 2018. The outbreak was suspected when the second case developed signs of IMD 2 months after the first case. Although the epidemiological link to Case 1 was clear, a strain was not identified for this second case. Case 3, who developed IMD 7 months after Case 2, had a microbiological profile identical to Case 1 and also had an epidemiological link, which confirmed the presence of an outbreak.

The aim of this report is to discuss the outbreak control measures taken and to highlight the specific issues of outbreak management that arose.

## Methods

### Case detection, investigation and outbreak management

All IMD cases as defined by the EU case definition [10] must be declared to the regional health authorities (Wallonia, Flanders and Brussels-Capital Region) as soon as suspected i.e. before confirmation. The regional authorities coordinate the response and implementation of control measures, as defined in regional guidelines [11,12]. Since this cluster was located in the Walloon Region, the case investigation, contact tracing and intervention were carried out by Wallonia’s infection prevention and control unit (Agence pour une Vie de Qualité (AViQ)). In the small rural school where the cluster occurred, two classrooms were affected, which included 50 children and two teachers.

Demographic data, data on clinical presentation, vaccination status, travel history, outcome and complications about each IMD case were collected by health officials through a standard telephone questionnaire with the clinicians and the parents, since the cases were under 18 years of age. An active search for close contacts of each case up to 7 days before the onset of symptoms was conducted in order to give chemoprophylaxis as quickly as possible to these contacts, i.e. within 24 to 48 h.

Close contacts were defined as follows: (i) household contacts, (ii) people who have had intimate or close contact with the case, e.g. kissing exchange and repeated physical contact, (iii) pupils in the same classroom and teachers in a preschool setting, (iv) children in the same nursery and (v) medical personnel who have performed a close-contact procedure, e.g. resuscitation manoeuver or intubation, etc.

In case of refusal of prophylaxis in a collective environment, e.g. school or creche, an exclusion of 7 days from the last contact with the index case is required. As defined in the regional guidelines, if the antibiotic administered to the patient as treatment is not a third generation cephalosporin (ceftriaxone, cefotaxime), an antibiotic eliminating pharyngeal carriage is administered. For serogroup A, C, W or Y, post-exposure vaccination is recommended when two cases of the same serogroup occur within a period of 1 month within a community. Sampling for carriage of close contacts is not part of standard IMD management in Belgium [11,12].

Primary clinical samples (taken before the antibiotic treatment is given), e.g. cerebrospinal fluid (CSF) and/or isolated *N. meningitidis* strains, were sent to the Belgian National Reference Centre (NRC) for *N. meningitidis* for confirmation, serogrouping and antimicrobial susceptibility testing. For the three cases, CSF was extracted via lumbar puncture and for Cases 1 and 3, blood cultures were also performed.

### Microbiological investigation

The bacterial isolates from CSF or blood were grown at 37 °C on Columbia Sheep Agar (Oxoid, Dilbeek, Belgium) in a candle air-exhaustion chamber. Serogrouping was performed by slide agglutination with Difco *Neisseria meningitidis* antisera (Becton Dickinson, Grayson, United States (US)). The minimum inhibitory concentrations (MICs) of penicillin G, cefotaxime, chloramphenicol, rifampin, azithromycin and ciprofloxacin were determined by ETEST (bioMerieux, La Balme, France). ETESTs were carried out according to the manufacturer's instructions, and the EUCAST breakpoint definitions [13] were used for the interpretation of antimicrobial susceptibility test results.

### Genomic investigation

Genomic DNA from pure bacterial culture was obtained using MagCore Genomic DNA Bacterial Kit (RBC Bioscience, New Taipei City, Taiwan) according to the manufacturer’s instructions. Nextera sequencing libraries were prepared and sequenced on an Illumina MiSeq (Illumina Inc., San Diego, California, US) using MiSeq Reagent Kit v3 (Illumina Inc.), obtaining 300 bp paired-end reads. Data were submitted to the European Nucleotide Archive (ENA; reference: PRJEB46584, ERS6675395 and ERS6675396). Data were analysed using an in-house pipeline [14] and the Neisseria PubMLST database (http://pubmlst.org) [15].

To find associated cases within the Walloon Region or elsewhere in Belgium, a comparison of core genome multilocus sequence typing (*N. meningitidis* cgMLST v1.0) [16] profiles of the strains in this outbreak with the NRC strain collection was performed using BioNumerics 7.5 (bioMerieux) with a categorical unweighted pair group method with arithmetic mean (UPGMA) clustering and default settings.

Comparison with the international *Neisseria* PubMLST database was performed by using the online available Bacterial Isolate Genomic Sequence database (BIGSdb) database and Genome Comparator tool [15].

### Ethical statement

All data accessed in the context of the present study had not been collected for research purposes but as part of the routine data collection for epidemiological surveillance, as stated in the Public Register dated 25/04/1997. In accordance with §9 of the latter authorisation and article 6, §1, e of the General Data Protection Regulation, no written informed consent from the patients is required for the collection and analysis of epidemiological data and treatment success collected when the processing of personal data is necessary for the performance of a task carried out in the public (health) interest.

## Results

### Outbreak outline

The outbreak occurred in the south of Belgium. The community has one local school and is characterised by isolated economic and social spheres. Before the outbreak, the three cases were healthy, attended the nursery school daily, and were vaccinated against MenC but not MenB. 

Case 1 was hospitalised in the intensive care unit with purpura fulminans in spring 2018. The diagnosis of meningococcal infection was made within 24 h and the case was notified to the health authorities the day after the hospitalisation. The meningococcal strain from the blood culture was sent to the NRC. Two months later, Case 2, who attended the same classroom as Case 1, developed symptoms typical of meningitis and was hospitalised. Lumbar puncture was performed; CSF culture was negative, but the PCR performed on CSF was positive for MenB. Unfortunately, there was insufficient CSF available for further subtyping analyses in the NRC and, therefore, no possibility of confirming a microbiological link with Case 1.

The third case occurred 7 months later, i.e. 9 months after Case 1. Case 3 attended another classroom but was in the same age group as the other two cases; notably, a sibling of one case was in the same classroom. As described in the Walloon guidelines, no follow-up sampling on carrier status was performed to confirm/disconfirm the hypothesis of potential subject in the transmission to Case 3. The three cases stayed in the hospital between 10 and 11 days and in the ICU between 3 and 8 days. All three cases recovered but one case presented with sequelae. More clinical and microbiological information about the three cases are available in the Table.

**Table:** Summary of clinical and laboratory information on three cases of invasive meningococcal disease serotype B, Wallonia, Belgium, 2018 (n = 3)

Characteristics	Case 1	Case 2	Case 3
Medical history	Previously healthy	Previously healthy	Previously healthy
Chemoprophylaxis^a^	NA	Spring 2018	Spring and summer 2018
Clinical presentation	Meningitis and sepsis (purpura fulminans)	Meningitis and sepsis	Meningitis and sepsis
Treatment^b^	Cefotaxime	Cefotaxime	Cefotaxime
Complications	Respiratory insufficiency, acute renal failure, disseminated intravascular coagulation	Septic shock	Moderate septic shock^d^, disseminated intravascular coagulation
Microbiology^c^			
Blood culture	MenB cc 269	NA	MenB cc 269
Cerebrospinal fluid PCR	Negative	Positive	Negative

cc: clonal complex; MenB: meningococcus B; NA: not applicable.

^a^ Chemoprophylaxis treatment was a single dose of ciprofloxacine (15 mg/kg).

^b^ Cefotaxime treatment (200 mg/kg/day) was administered until the case was fully recovered.

^c^ Strains had identical antibiotic sensitivity profiles, including absence of fluoroquinolone resistance.

^d^ We followed the international definition of moderate septic shock [34].

All cases were between 3 and 5 years of age and attended the same school.

### Microbiological analyses

For Case 1 and 3, microbiological analyses for finetyping revealed a MenB with identical genotype for Case 1 and 3, according to international standards (PorA VR1: 5-1; PorA VR2: 2-2; FetA: F5-1; clonal complex: cc269). Both strains were susceptible to all tested antibiotics, including absence of fluoroquinolone resistance. Neighbour-net phylogenetic network analysis, which was based on a comparison of 1,605 core genes of the Neisseria cgMLST v1.0 typing scheme using BIGSdb genome comparator tool, confirmed the two cases as nearly identical (only five allele differences among 1,605 loci). The 4CMenB vaccine strain coverage was predicted based on whole genome sequence data according to genomic Meningococcal Antigen Typing System (gMATS) [17], although only by antigenic cross-reactivity since the strain presented factor H binding protein (fHbp) peptide variant 15 and neisserial heparin binding antigen (NHBA) variant 21.

For Case 2, no strain could be cultured, and thus no finetyping was performed. The case was subsequently confirmed as MenB, but only by PCR on cerebrospinal fluid in the hospital laboratory.

### Genomic investigation

We identified three other IMD cases in 2016 and one in 2017 with related isolates (< 50 cgMLST allele differences) in the same Walloon province.

Comparison with the international PubMLST database revealed four more distantly related strains (63–88 allele differences) isolated in France during the period 2014–16. This indicates a genetic lineage present at a low level in this geographical region for some years. The genetic comparison of the cluster strains with the international database is shown in the Figure.

**Figure:**
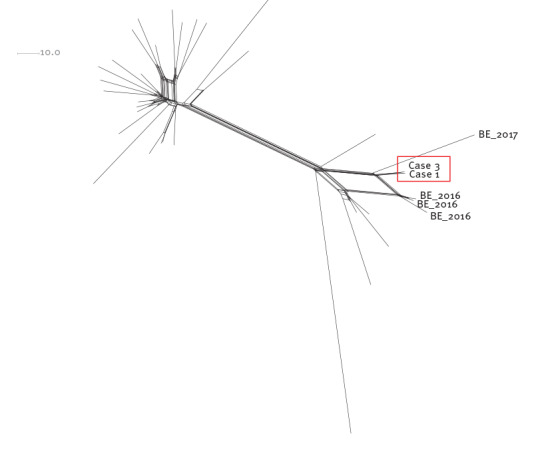
Genomic comparison of meningococcus B strains, Wallonia, Belgium, 2016–2018 (n = 6 strains) and EU/EEA, 1999–2019 (n = 30 strains)

BE: Belgian isolate; EU/EEA: European Union/European Economic Area.

The Neighbour-net phylogenetic network based on a comparison of 1,605 core genes of the Neisseria cgMLST v1.0 typing scheme among a subset of genomes with less than 200 allele differences with the cluster cases of all publicly available genomes using BIGSdb genome comparator tool: the outbreak cluster strains (n = 2 cases; red box), historical Belgian cases (n = 4 cases; BE and year of identification) and internationally-related cases (n = 30 cases). All related BE cases (< 50 cgMLST allele differences) were observed in the same Walloon province. Neighbour-net graph visualised using SplitsTree4.15.1 [35].

## Outbreak control measures

After Case 1 and Case 2 occurred, ciprofloxacin chemoprophylaxis (a single dose of 15 mg/kg) was administered to all close contacts of Cases 1 and 2 within 48 h, i.e. household contacts including parents and siblings, children in the class and the teachers, by the health authorities. Since there were no recent activities outside the classroom group and since the teachers were exclusively in charge of only one group of children, ca 35 individuals received chemoprophylaxis per case. Of note, all close contacts completed the treatment course.

As for Cases 1 and 2, chemoprophylaxis was again administered to the close contacts of Case 3 after MenB diagnosis, at the end of 2018. Since there were activities with other classes of older students (aged 6–7 years), chemoprophylaxis was also given to these students (total n = 150 in the school) with 100% uptake. Azithromycin (a single dose of 10 mg/kg) was administered instead for those sceptical about the effectiveness of ciprofloxacin.

After the third case, the need to recommend vaccination against MenB for the other children in the classroom was discussed with Belgian infectious diseases experts. In Belgium, at the time of the outbreak, guidelines about vaccination for MenB outbreaks were only available in the Flanders region; with the occurrence of a cluster caused by serogroup B, MenB vaccination was recommended for contacts who received chemoprophylaxis and were not vaccinated against MenB within the last 12 months [12]. The pros and cons of offering vaccination with 4CMenB to contacts were evaluated (summarised in the Box).

Box **Pros and cons of offering vaccination to close contacts of cases with invasive meningococcal disease serotype B**

**Table:** 

Pros	• Vaccination is the only existing long-term protection, as mass chemoprophylaxis is a temporary protective measure [28]. • The strain of this outbreak is covered by the 4CMenB vaccine.
Cons	• The vaccine Men4B is not considered as a standard intervention in case of outbreak in Wallonia. • The cost of the vaccine, administered in two doses and not reimbursed in Belgium, is not negligible. • The most at-risk period for meningococcal infections is between ages 0–5 years [1-3], so the children affected by this cluster were in the process of exiting this risk period. • There is no evidence of a decrease in the prevalence of post-vaccination carriage of the strain during an outbreak in the population, so the vaccine is unlikely to provide herd immunity in the context of an outbreak response [20,21].

Pros and cons were outlined following a discussion with Belgian infectious diseases experts after the third case of MenB in a nursery school within a 9-month period.

The decision to recommend 4CMenB vaccination to the two nursery school classes was made ca 1 month after Case 3 became ill. The final choice to vaccinate was left at the discretion of the parents. The vaccinations (two doses) could be administered either by the school doctor, general practitioners and paediatricians. It was decided that 4CMenB vaccination status would remain confidential so the number of actual vaccinations given after the outbreak is unknown. Of note, the cost for vaccination was partially reimbursed by the school and the school health promotion service (whether administered by the school doctor or a doctor outside the school); reimbursement was requested for 40 of the 50 children. In the 12-month follow-up after Case 3, no additional cases were reported by the nursery school.

## Discussion

We describe here a prolonged outbreak of three cases with IMD serogroup B which occurred between spring and the end of 2018. The epidemiological link and the nearly identical cgMLST profile of both strains of Cases 1 and 3 strongly indicates that these two cases are linked.

Most of the IMD outbreaks described in the literature concern two or three cases and occur within 21 days – often during the first week – following the first case [7-9]. The 9-month duration of this outbreak in a nursery school, with intervals of 2 and 7 months between cases, is quite uncommon. Prolonged outbreaks of IMD have already been described, especially with MenB, in a geographically limited area (community-based outbreaks) [18,19] and in an institution, especially universities in the US [20-22]. However, IMD outbreaks are rarely described in preschool or school settings. A prolonged outbreak with five cases of MenB over a 4-month period has been described in a nursery in England in 2013 [23]. In the United Kingdom (UK), children aged 1–4 years are particularly affected and the relative risk of further cases in the 4 weeks after a single case is 27.6 (95% CI: 15.2–39.9) in preschool settings [8,9]. In the US, people aged 11–24 years are more likely to be affected and universities represent 47.1% of organisation-based clusters [24].

The clonal complex cc269, as described in this report, has been described as a hyper-virulent lineage. Strains belonging to this lineage have been responsible for outbreaks both in Belgium and worldwide [25,26]. Meningococci are genetically very diverse and evolve rapidly due to horizontal gene transfers. In order to correctly identify outbreaks, it is important to interpret genome distances and their phylogenetic relationship compared with other sequenced strains not from outbreaks [27]. Among the over 300 IMD cases reported in Belgium since 2016, the six IMD isolates described in this case report are the only ones that fall within 50 cgMLST allele differences. All cases were from the same Walloon province, suggesting that there is probably substantial carriage of this lineage of related strains in this particular region. While Belgium is a small country, it has a high density and sustains high human movement, both domestic and international; our data suggest that specific clones are very local, and persist for several years. According to the global pubMLST database, related strains have only been observed in France, which also confirms this narrow geographical representation. However, whole genome sequencing results show a clear difference in genetic distance between the cluster cases (five allele differences) and historical IMD cases (20–45 allele differences), indicating active circulation of this particular strain in this school setting during the timeframe of the outbreak. Reintroduction could have occurred by an asymptomatic carrier among those around young children in the school who had not received antibiotic prophylaxis or who had been recolonised, rather than local circulation of the lineage of related strains.

The purpose of chemoprophylaxis is to eliminate carriage of *N. meningitidis* before the bacterium causes invasive disease or is transmitted to others. Mass chemoprophylaxis in response to an outbreak is associated with a considerable reduction of meningococcal carriage [28]. This protection is temporary, i.e. for several weeks, but in most outbreaks, no further cases appear after mass chemoprophylaxis. The antibiotic of choice was ciprofloxacin, according to the regional guidelines [11], for which the outbreak strain confirmed to be susceptible. After reoccurrence with the third case, chemoprophylaxis was changed to azithromycin, for which the outbreak strain also confirmed to be susceptible. According to ECDC guidelines [29], both antibiotics are advised for chemoprophylaxis and the switch was rather made out of precaution and for general perception of the impacted close contacts. The reduction in carriage is often effective if the antibiotic coverage is wide, i.e. greater than 90% [28], which was the situation in this outbreak. This raises the question about the need of additional measures like vaccination and carriage screening in the management of a cluster.

One difficulty in the management of this outbreak was taking the decision to recommend mass vaccination in the school classes. Since the vaccines 4CMenB and rLP2086 became available in Europe in 2013, mass vaccination has frequently been organised in MenB outbreak situations [8,20,21,24,30]. In England, although new cases were detected after chemoprophylaxis during outbreaks, which was because of the different meningococcal serogroups; no cases occurred after vaccination [8]. In the US, among the 10 MenB-related outbreaks in universities between 2013 and 2018, five still presented additional cases after a vaccination campaign (with rLP2086 or 4CMenB), probably as a result of low vaccination coverage and the absence of herd immunity linked to these vaccines [22]. US, France and UK recommend vaccination against MenB for the management of outbreaks, according to certain criteria [31-33]. France recommends vaccination in an outbreak of two or more cases in the same community or social group within a maximum period of 3 months [33]. The UK recommends vaccination against MenB to the same group that would receive antibiotic chemoprophylaxis [32]. In the US, where prolonged outbreaks related to MenB – in universities, in particular – have had to be managed on several occasions [21,22,24], the US Centers for Disease Control and Prevention (CDC) gives indicative thresholds for deciding the need for vaccination. However, these thresholds are flexible and each situation must be considered on a case-by-case basis [31].

At the time of this outbreak, the Wallonian regional guidelines did not recommend post-exposure vaccination for MenB. This outbreak showed that even with administration of chemoprophylaxis, the strain can continue to circulate locally. While vaccination of close contacts could protect them from future infection, this outbreak showed the need to extend the coverage to close contacts in other classrooms, because of the particular social setting. Furthermore, the absence of an effect on herd immunity of the currently available MenB vaccines suggests that a wider range of vaccination should be emphasised. In this outbreak, the cost of the vaccine, which was not reimbursed in Belgium, was one of the cons against mass vaccination, although it is only a small part in the total cost for the management of an outbreak. Because of this outbreak, the Wallonian regional guidelines were adapted in January 2021 and now do recommend MenB vaccination of close contacts when two epidemiologically linked cases occur within 1 month [11]. However, this outbreak, which exceeded the 1-month period, highlights the need for flexible case-by-case measures depending on the social setting, especially when there is strong microbiological evidence.

### Limitations

This report has several limitations. The absence of a strain for the second case is unfortunate because it would have permitted confirmation that all the cases were linked. The fact that the second case had a positive PCR for meningococcus serogroup B and that this child attended the same classroom as the first case makes it very probable that there is a link to both Cases 1 and 3. Moreover, this outbreak occurred in a rural school and there was no contact between cases outside the school setting. Regarding the total number of MenB vaccinated children, we only know the number of children for whom reimbursement has been requested to the school medicine service. It is possible that more children may have been vaccinated via a general practitioner or paediatrician without a request for reimbursement.

## Conclusions

Despite contact tracing efforts, IMD outbreaks are difficult to manage and generate public anxiety, particularly in the case of an ongoing cluster. In such situations, use of MenB vaccines for post-exposure prophylaxis vaccination is a durable option to protect close contacts, but is poorly documented. Determining thresholds, i.e. number of cases, time period, target group size, remains a challenge and requires further investigation.

## Availability of data and materials

Raw reads of whole genome sequencing are available in ENA (reference PRJEB46584, ERS6675395 and ERS6675396).
